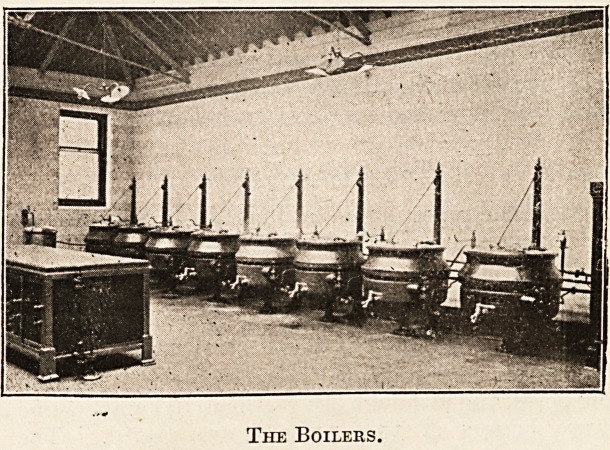# The Units of General Hospital Construction

**Published:** 1907-07-06

**Authors:** 


### HOSPITAL ADMINISTRATION.

### CONSTRUCTION AND ECONOMICS.

### THE UNITS OF GENERAL HOSPITAL CONSTRUCTION.

#### THE HOSPITAL KITCHEN.

THE situation of the kitchen and its relation to the other departments of the hospital are matters regarding which various opinions are held. Much depends on the area of the site available. Where this is limited, as is frequently the case in city hospitals, the buildings are necessarily more cramped, and every available foot of ground is utilised. If the site is sufficiently large a detached building is to be preferred, and its situation should be on the level of the basement or ground floor behind the central administrative buildings, or it may with advantage be connected with the hospital by a cross ventilated passage. When the ventilation of the kitchen is carried out by means of an extraction fan, and this cut-off passage separates it from the other parts of the institution, the corridors and the hospital generally should be as free from the undesirable odour of cooking as if the kitchen were on the top story. This situation behind the main buildings and adjacent to a central corridor in the basement running the full length of the hospital and connected with the different floors by lifts, facilitates the efficient and rapid service of hot food to the various units.

#### ECONOMISING STEAM.

It has been argued that the kitchen must be placed as near the steam boilers as possible, in order to economise steam. There is little to justify such a proposal, for if steam pipes are properly covered and carried in a duct from the boiler to the kitchen, the loss of steam is hardly worth taking into consideration. Calculations recently made give the following results: In an uncovered pipe, 120 ft. long and 6 in. in diameter, 1.3939 lb. of steam condenses per lineal foot per hour, when the pressure of steam in the boiler is 100 lb. and the external temperature 62° Fah. When the pipe is covered only .1747 lb. condenses under the same conditions. In a properly covered pipe, therefore, with a steam pressure of 100 lb., and assuming that 293 lb. of steam is passing through the pipe per minute, the loss is only 1 lb. pressure at a distance of 120 ft. Consequently the question of distance from the boilers has very little practical bearing on the situation of the kitchen, which must be governed by other and more important considerations.

#### POSITION OF THE KITCHEN.

The kitchen should, of course, be placed in the most central position, and where the site is limited in area the top flat of the central administrative block is much in favour. From here the odour of cooking does not permeate the other parts of the building, and possibly the limitations of the site may leave this the most convenient, and, under the circumstances, the most perfect position attainable. But there are disadvantages which must be considered. In the first place there is additional cost of construction, because in order to carry the heavy apparatus now universally adopted the walls and floors must be stronger than would otherwise be required. There is, further, the increased labour in service. All food must first be taken up to the kitchen, and taken down again for distribution to the various parts of the hospital. All fuel must be similarly conveyed, and all ashes, refuse, etc., taken down. The distance from the various stores (provisions, meat, milk, etc.) when these are on the basement floor is a disadvantage. Doubtless to a certain extent the resulting inconvenience can be modified by lifts, but where a detached basement site is available the advantages are all in favour of placing the kitchen there.

#### THE KITCHEN.

The size and shape of the kitchen when in a detached building can be made to suit the requirements, and are only governed by these requirements; not, as in top-storey kitchens, by architectural considerations of the understructure to which the kitchen must necessarily conform. When the size is out of proportion to the work to be overtaken, and the shape entails the placing of stoves, steam pans, and other fittings in inconvenient situations, the additional labour and time involved only means increase of staff without any compensating advantage. A kitchen 46 ft. long by 34 ft. wide, with a scullery adjoining, 26 ft. square, is large enough to serve for 700 to 800 persons.

THE BOILERS.

#### THE COOKING APPARATUS.

The kitchen of an up-to-date hospital will, of course, be fitted with the most modern ranges, stoves, boilers, and steamers, in order that the food may be thoroughly cooked with the least possible expenditure for fuel and the greatest economy of labour. There are so many excellent patterns of such fittings that it would be an invidious task to make any distinctions here. But many kitchens possessing such excellent fittings have them arranged in such a manner that much of the benefit which might otherwise result is negatived. Badly arranged fittings add to the difficulties in working and to the cost of service, and some general principles might with advantage be indicated here under this head.

### Fittings.

All fittings should stand at least 18 inches clear of the wall. No pipes should be hung on the wall, the steam and water pipes being supported on iron standards, with sufficient space to allow a workman to pass behind them should repairs be at any time necessary. All steam ovens, steam pans, tea infusers, indeed every fitting requiring a steam supply (except perhaps the hot plate or carving table) should be arranged along one side, which obviates multiplication of pipes. Where a range of steam pans is so placed the fitting of alternate hot and cold supply taps on a swivel effects a considerable saving in plumbing. Let us suppose there are eight steam pans. A hot water tap between one and two can, with a swivel mechanism, supply both. A similar cold water tap between two and three supplies both. A hot water tap is then placed between three and four, and a cold water tap between four and five, and so on. The first and last pans have a cold water tap on their outer side. Thus eight pans have a supply of both hot and cold water with nine taps, instead of the sixteen which would be required were each furnished with its own fixtures.

### The Steam Pipes.

All steam pipes should be flanged and not screwed at the joints, a decided advantage being thus secured in the event of leakage. Screw-down taps for hot and cold water are also to be preferred. Many firms specialise in boiler construction and claim advantages for particular patterns of boilers. Tilting boilers are advantageous for emptying and cleaning purposes, and although the gear may be cumbersome large boilers of this pattern are useful. The objection to the spiket type is the difficulty of cleaning that form of outlet, but it can be quite satisfactorily overcome by having a screw on the outlet level large enough to admit a brush which can clean it thoroughly. Whatever variety is employed, however, it should be jacketed only half-way up. Completely jacketed boilers are liable to cook the contents more rapidly at the top than at the bottom, and boiling over means probably the premature emptying of the pan. Those who have worked with a metal band on the boiler lid will appreciate the advantages of a vulcanite handle on the side of the lid, which is not only handier, but less dangerous in use.

Some hospital authorities provide no coal range, but depend entirely on steam and gas cookers. This, of course, obviates the necessity of coal supply and removal of ashes, but many cooks prefer the coal range with roasting ovens for special purposes, such as the roasting of butcher's meat or the baking of puddings. Gas ovens will not "brown" fish or a pudding so effectively. But the old objections to gas cookers have now almost entirely disappeared, and if a reliable firm is chosen and a first-class article secured, gas apparatus is found to be useful and economical.

All furnishings, such as dressers and racks for pots and pans, should be on wheels, in order that they may be regularly drawn out from the wall to facilitate cleaning. This avoids any possibility of dirt or vermin collecting. The scullery adjoining the kitchen should be supplied not only with the usual range of sinks for various purposes, such as cleaning vegetables, potatoes, pots, pans, etc., but, in addition, with a special sink or washhand basin for the use of the maids. This is a point too frequently overlooked.

### Trolleys.

Another advantage is the provision of trolleys to hold the trays for the fish and potato steamers. By this means the trays of potatoes, vegetables, etc., are wheeled to the cookers, representing an immense saving in labour. It will also be found advantageous to have a specially designed wheeled box under the potato and vegetable sinks, which acts as a receptacle for all sand and dirt. The contents of the sink run into this box, in the centre of which is placed an upright, perforated tube. Through this the water flows into the soil pipe, the solids being retained in the bottom of the box, from which they can be removed. Those who have experienced the choking of drains with mixtures of grease and sand can appreciate the advantage of this device.

### The Kitchen Floor.

The floor of the kitchen should be made of some non-absorbent material, the best being Ruabon tiles, which are comparatively cheap, look clean and tidy, and never get greasy or stained. There should be a slight fall on the floor to the side wall, where a shallow gutter should be formed in concrete. Walls should be covered with a hard glazed tile up to the roof. The lighting should be partly from the roof, and the roof light should be principally from the north. Adjoining the kitchen special larders should be provided for milk, vegetables, dripping, cold meats, preparation of pastries, etc. (in Scotch hospitals bread is not baked in the hospital, and therefore a large bakery is not provided), and a housekeeper's store room. The other stores will be dealt with under the storekeeper's department.

The management of the kitchen is carried out by a lady, who should have a complete training in housekeeping and cookery, and be capable not only of supervising the whole of the domestic department, but of giving lectures and demonstrations to nurses on sick-room cookery and household management generally. In addition to the supervising housekeeper, the staff should consist of a head cook, a boiler maid, maid for the coal range, and assistants. All the staff should have their quarters as near the kitchen as possible. The housekeeper's private rooms should be in the same unit, so that all her staff are under her direct supervision.

## Figures and Tables

**Figure f1:**